# Clinical study of early rehabilitation training combined with negative pressure wound therapy for the treatment of deep partial-thickness hand burns

**DOI:** 10.3389/fsurg.2023.1040407

**Published:** 2023-02-08

**Authors:** Canbin Liu, Hongteng Xie, Pei Wei, Teng Gong, Guohua Wu, Zhaorong Xu, Shun Chen

**Affiliations:** ^1^ Burn & Wound Repair Department, Fujian Medical University Union Hospital, Fuzhou, China; ^2^ Fujian Burn Institute, Fujian Medical University Union Hospital, Fuzhou, China; ^3^ Fujian Burn Medical Center, Fujian Medical University Union Hospital, Fuzhou, China; ^4^ Fujian Provincial Key Laboratory of Burn and Trauma, Fujian Medical University Union Hospital, Fuzhou, China

**Keywords:** hand burn, negative pressure wound therapy, total active motion, brief Michigan Hand Function profiles, early rehabilitation training

## Abstract

**Objective:**

This study aims to explore the clinical effect of early rehabilitation training combined with negative pressure wound therapy (NPWT) for treating deep partial-thickness hand burns.

**Methods:**

Twenty patients with deep partial-thickness hand burns were randomly divided into an experimental group (*n* = 10) and a control group (*n* = 10). In the experimental group, early rehabilitation training combined with NPWT was performed, including the proper sealing of the negative pressure device, intraoperative plastic brace, early postoperative exercise therapy during negative pressure treatment, and intraoperative and postoperative body positioning. Routine NPWT was conducted in the control group. Both groups received 4 weeks of rehabilitation after wounds healed by NPWT with or without skin grafts. Hand function was evaluated after wound healing and 4 weeks after rehabilitation, including hand joint total active motion (TAM) and the brief Michigan Hand Questionnaire (bMHQ).

**Results:**

Twenty patients were involved in this study, including 16 men and 4 women, aged 18–70 years, and the hand burn area ranged from 0.5% to 2% of the total body surface area (TBSA). There was no significant difference in TAM and bMHQ scores between the two groups after negative pressure removal. After 4 weeks of rehabilitation training, the TAM scores and bMHQ scores were significantly improved in both groups (*p* < 0.05); among them, those of the experimental group were both significantly better than those of the control group (*p* < 0.05).

**Conclusion:**

The application of early rehabilitation training combined with NPWT to treat deep partial-thickness hand burns can effectively improve hand function.

## 1. Introduction

The hands are one of the most important parts of the human body. Although the hands only account for 3%–5% of the surface area of the body, they are especially vulnerable to injury (1). Deep partial-thickness hand burns that require surgery account for approximately 40% of hand burns. Negative pressure wound therapy (NPWT) is a safe and effective method for the treatment of hand burns, which promotes wound repair by accelerating angiogenesis and cell proliferation, contracting wound, reducing edema, and removing exudation (2, 3).

Despite improvement of surgical therapy for deep partial-thickness hand burns, the contracture deformity and dysfunction caused by scar formation still reach 50%–70%, making the hand the most frequent site for reconstructive surgery due to scar contracture within 10 years after a burn. The effective treatment of deep partial-thickness hand burns requires multidisciplinary concepts and technologies such as burn centers, plastic surgery, rehabilitation, and physical therapy, and early and precise treatment is the basis for the best recovery of hand function (4, 5).

Early recovery and training of patients, especially critical care patients, have been proven to be safe, effective, and feasible treatments for improving prognosis (6–10). Therefore, early rehabilitation training has received more attention and is accepted by medical workers. However, according to an investigation of the current situation of burn rehabilitation in mainland China, traditional rehabilitation training for burn patients is only offered after the wound has healed (11) when some patients have already suffered functional limitations. Therefore, on the basis of the NPWT treatment experience of deep partial-thickness hand burns in our department in recent years, we believe that the application of early rehabilitation training combined with NPWT could effectively improve hand function in the treatment of deep partial-thickness hand burns. In this study, we introduced early rehabilitation training combined with NPWT for treating deep partial-thickness hand burns and evaluated its clinical effect.

## 2. Materials and methods

### 2.1. Subjects of the study

This study was a prospective, randomized, single-center trial. Twenty patients (20 injured hands) with deep partial-thickness hand burns admitted to Fujian Medical University Union Hospital from March 2019 to December 2021 were selected. The admitted patients met the following criteria: (1) patients’ age ≥18 years; (2) patients with deep partial-thickness hand burns, even if they were complicated with other body parts being burned, as long as this did not affect the treatment of the hand wounds; (3) they could be complicated with other diseases as long as this did not affect the treatment of the hand wounds; and (4) patients who agreed to participate in the study and signed the informed consent. Exclusion criteria was as follows: (1) those who could not cooperate with the experiment; (2) those with mental and physical disabilities; and (3) those who had other serious diseases that affected the treatment of the wound.

The patients were randomly divided into groups. First, they were given a series of labels 1–20 according to the order of their coming to the hospital. A random number was then generated for each label using SPSS 26.0. The patients were grouped according to the size of their assigned random numbers. Patients with the 10 largest numbers were specified as the control group, and the others were assigned to the experimental group.

### 2.2. Treatment methods

After admission, debridement was conducted under anesthesia, and the dead skin and any foreign bodies were removed. The wounds were rinsed with iodine and normal saline, followed by covering with a vacuum-sealed drainage kit (Wuhan Weisidi Medical & Technology Co., Ltd.). The negative pressure foam was shaped to fill between the fingers, and the edges of the foam were sutured to the wound edge to ensure that the wound was fully covered without leaving any space. The wound was sealed with polyurethane film, and a drainage tube was connected to the negative pressure device maintained from 100 to 120 mmHg for continuous negative pressure suction. The negative pressure device was changed, and the wound was assessed every 5–7 days. Then, it was decided whether skin grafting could be conducted. The patient's hand was immobilized for 5 days after the operation if a skin graft was conducted.

**Figure 1:**
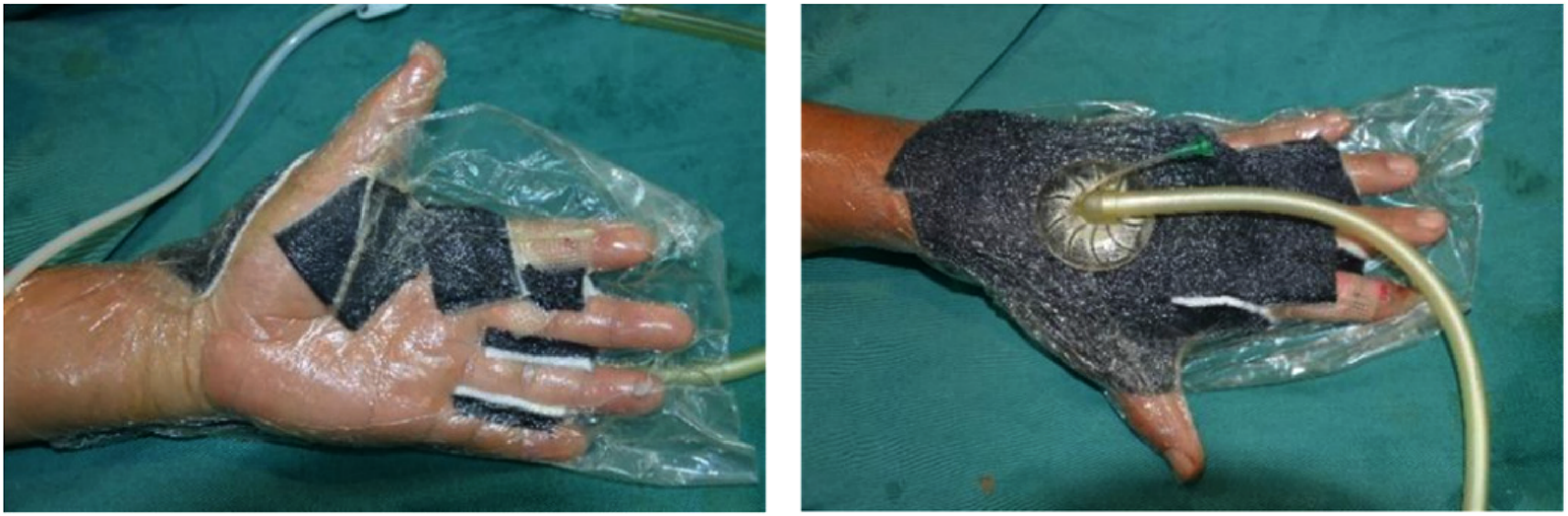
During NPWT, the interphalangeal negative pressure material was properly placed and the thumb was kept in abduction to allow for postoperative rehabilitation as soon as possible. NPWT, negative pressure wound therapy.

**Figure 2:**
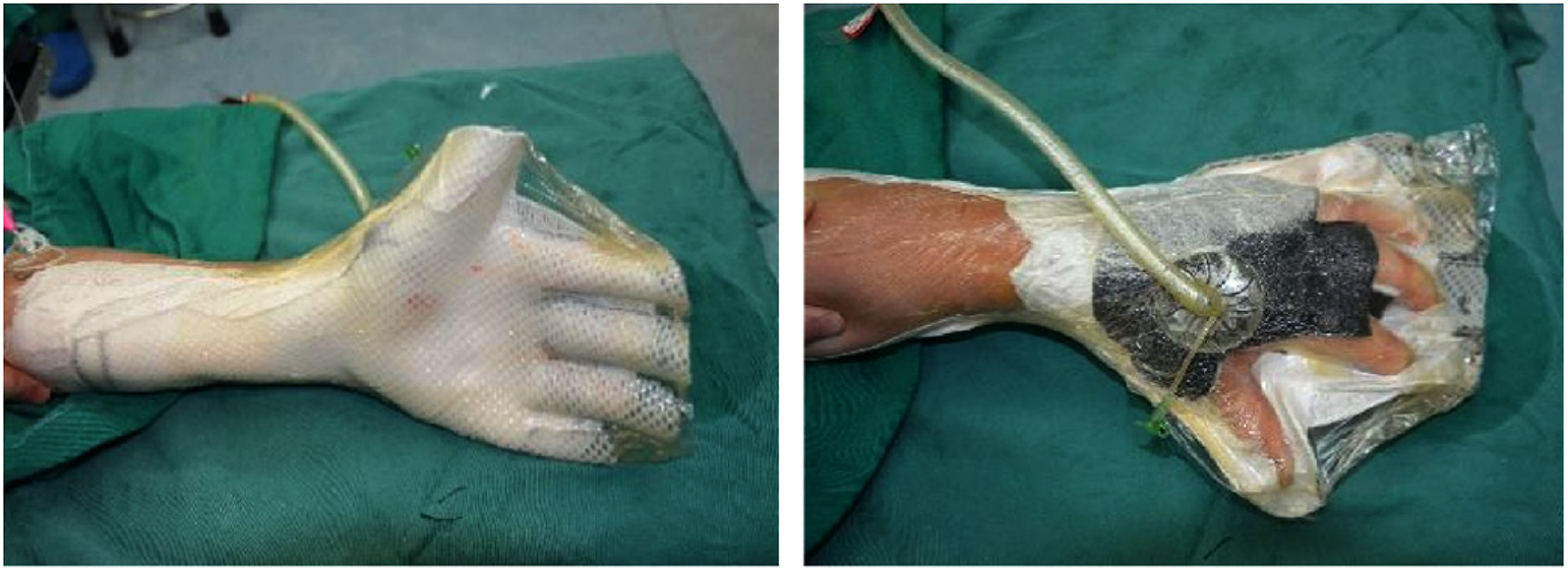
With skin graft, a plastic brace was used to keep the injured hand in the functional position.

**Figure 3:**
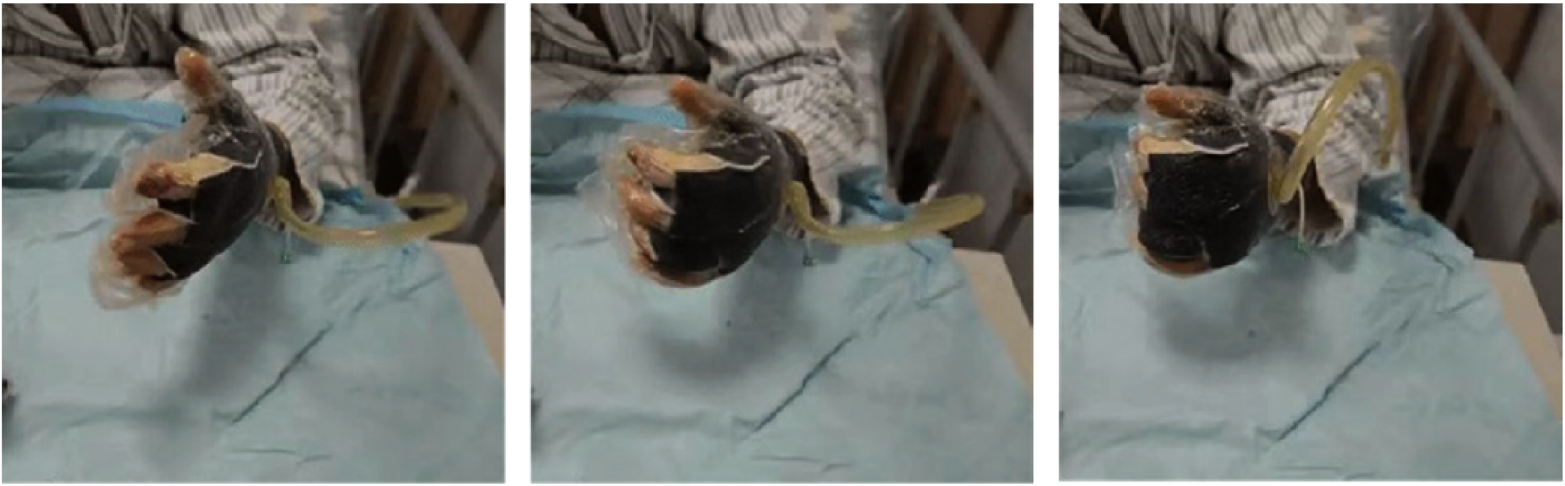
Without skin graft, the patient was guided to undergo early rehabilitation training at the bedside while under negative pressure treatment.

In the experimental group, early rehabilitation training combined with NPWT was performed. The key point was that the negative pressure foam between the forefinger and thumb was positioned to ensure that the thumb could abduct as much as possible (Figure 1). (1) When skin grafting was needed, a brace made of low-temperature plastic material was used to keep the hand in a functional position after skin grafting (Figure 2), and the limb was kept raised 10°–30°after surgery. During immobilization, the patients were encouraged to perform isometric (static) contractions to improve their muscle strength and reduce edema. (2) If a skin graft was not conducted, patients were instructed to carry out finger joint motion and muscle strength training during the early postoperative stage until the negative pressure device was removed (Figure 3).

After the wound healed, both groups began routine rehabilitation treatment, including pressure therapy, exercise therapy (active and passive movement of the finger joints, 1–2 times/day, 10–20 min/time), occupational therapy, upper limb intelligent motor feedback training (1 time/day, 10–20 min/time), scar massage, and bracing at the functional part. The hand brace was adjusted or replaced in a timely manner to meet the functional requirements of the patients at different stages.

### 2.3. Measurement and assessment

#### 2.3.1. Total active motion

The total active motion (TAM) of each finger joint of all 20 patients was measured by a joint angle ruler after wound healing by NPWT with or without skin grafting and again at 4 weeks after rehabilitation. The TAM system assessment method recommended by the American Society for Surgery of the Hand (12, 13) was adopted. The sum of joint motion (TAM) of the maetacarpophalangeal joint (MP), proximal interphalangeal joint (PIP), and distal interphalangeal joint (DIP) was compared with the healthy values.

The excellent and good rates of hand function were also assessed. The TAM score equal to 100% of the healthy side is considered excellent; the TAM score greater than or equal to 75% and less than 100% of the healthy side is considered good; the TAM score greater than or equal to 50% and less than 75% of the healthy side is considered fair; the TAM score less than 50% of the healthy side is considered poor. The excellent and good rate of hand function recovery = (excellent + good)/total number of injured hands × 100%; the qualified rate = (excellent + good + fair)/total number of injured hands × 100%.

#### 2.3.2. Brief Michigan Hand Outcomes Questionnaire

The brief Michigan Hand Questionnaire (bMHQ) was developed by the University of Michigan in 2011 based on the full version of the MHQ and had a high correlation with the full version of the MHQ, including six health domains (overall hand function, activities of daily living, pain, work performance, esthetics, and patient satisfaction) and 12 questions, which can be used as an important tool to measure the patient's prognosis and the quality of hand surgical care (14). The bMHQ results of this study were converted into a percentile system.

### 2.4. Statistical processing

SPSS 26.0 was used to analyze the data of this study, with the statistical significance level set to *p* < 0.05. The mean ± SD was used to record the measurement data. The dataset was tested for normal distribution with the Shapiro–Wilk test. The homogeneity of variances and covariances was tested and discussed, then an independent sample *t*-test or *t*’-test was used. Qualitative variables were reported as numbers and percentages, and the chi-square test was used.

## 3. Results

### 3.1. Patient characteristics

Twenty patients (20 injured hands) with deep partial-thickness hand burns were selected in this study, including 16 men and 4 women. Their age ranged from 18 to 70 years, and the hand burn area ranged from 0.5% to 2% TBSA, including 6 cases of scalding and 14 cases of flame burn. The patients were randomly divided into groups. There were seven men and three women in the control group, who were 42.2 ± 12.18 years old. There were eight men and two women in the experimental group, who were 42 ± 15.31 years old. There was no significant difference in age, sex, or cause of injury between the two groups.

### 3.2. TAM score

There was no significant difference in the TAM score after wound healing with or without skin grafting by NPWT (*p* > 0.05). After 4 weeks of rehabilitation, the TAM scores in both groups improved, and the differences were statistically significant (*p* < 0.05). Furthermore, the TAM score in the experimental group was significantly higher than that in the control group, and the difference was statistically significant (*p* < 0.05) (Table 1).

**Table 1:** Comparison of TAM scores between the two groups $\left(\bar{x}\pm s\right)$.

Group	Number of cases	Wound healed by NPWT with or without a skin graft	After 4 weeks of rehabilitation
Control group	10	112.26 ± 33.38	155.2 ± 39.04^a^
Experimental group	10	119.24 ± 35.23	175.58 ± 42.69^a,b^

NPWT, negative pressure wound therapy; TAM, total active motion.

^a^ After 4 weeks of rehabilitation, the TAM scores were significantly improved compared with when the wounds were healed by NPWT with or without skin grafts (*p* < 0.05).

^b^ TAM score in the experimental group was significantly higher than that in the control group (*p* < 0.05).

### 3.3. Comparison of the excellent and good qualified rates

The qualified rates were 100%, and the excellent and good rates were 60% and 10%, respectively, after 4 weeks of rehabilitation. The excellent and good rates of the experimental group were higher than those of the control group, and the difference was statistically significant (*p* < 0.05) (Table 2).

**Table 2:** Comparison of excellent, good, and qualified rates (%).

Group	Number of cases	Excellent	Good	Fair	Poor	Good and excellent rate	Qualified rate
Control group	10	0	1	9	0	10	100
Experimental group	10	1	5	4	0	60^a^	100

^a^ The experimental group was significantly higher than the control group (*p* < 0.05).

### 3.4. Comparison of the bMHQ scores

There was no significant difference in bMHQ scores between the two groups after wound healing by NPWT with or without skin grafting (*p* *>* 0.05). After 4 weeks of rehabilitation, the bMHQ scores of both groups were significantly improved, and the difference was statistically significant (*p* < 0.05). The bMHQ score of the experimental group was significantly higher than that of the control group, and the difference was statistically significant (*p* < 0.05) (Table 3).

**Table 3:** Comparison of bMHQ scores $\left(\bar{x}\pm s\right)$.

Group	Number of cases	Wound healed by NPWT with or without a skin graft	After 4 weeks of rehabilitation
Control group	10	37.66 ± 5.33	67.16 ± 5.52^a^
Experimental group	10	41.16 ± 5.68	76.16 ± 4.94^a,b^

NPWT, negative pressure wound therapy; bMHQ, brief Michigan Hand Questionnaire.

^a^ After 4 weeks of rehabilitation, the bMHQ scores were significantly improved compared with when the wounds were healed by NPWT with or without skin grafts (*p* < 0.05).

^b^ bMHQ score in the experimental group was significantly higher than that in the control group (*p* < 0.05).

## 4. Discussion

According to the International Society for Burn Injuries, approximately half a million burn patients need medical service each year, with 39% of burns involving the upper limbs and hands (15, 16). Although the mortality rate of hand burns is very low, the disability rate is very high. Scar contraction can severely affect hand function and may even require reconstructive surgery (17, 18). Therefore, active early treatment of hand burns is very important, including debridement, skin grafting, edema prevention, orthosis, and early rehabilitation training (19). Studies have shown that acute development of burn scar contraction can be avoided among burn survivors who receive adequate care and rehabilitation in hospitals. Methods of burn rehabilitation include psychological intervention, compression therapy, bracing, functional exercise, immersion therapy, etc. (20–22), among which early rehabilitation training is an important part of the whole sequence of treatment.

Early burn rehabilitation is generally defined as rehabilitation management that begins within 7 days after a burn injury. However, the concept of early rehabilitation training is still not widely accepted in China; only 29% of burn centers initiate early rehabilitation within 1 week after a burn injury, and these are mainly concentrated in severely burned patients (8–11, 23). For severe hand burns, routine rehabilitation usually only starts after the wound is healed, but the consensus is to start training as soon as possible after traditional hand surgery. Some scholars have proposed that early active tendon mobilization can be started during surgery or within 5 days after surgery (24, 25). Therefore, how to carry out early rehabilitation in the treatment of deep hand burns, how early to start, and how to implement them were the starting points of our research.

NPWT can increase tissue blood perfusion and reduce local edema to benefit deep burn wounds, which can not only promote wound repair but also shorten the finger immobilization time and realize early rehabilitation through early wound healing and reduction of complications, thus preventing secondary deformities and achieving successful functional recovery (26). However, in our clinical work, we found that during routine NPWT, because the fingers are fixed by foam dressings, adequate exercise training cannot be carried out, which prevents full rehabilitation and causes a certain degree of postoperative stiffness.

Therefore, our research extended rehabilitation therapy to the early stage of deep partial-thickness hand burn treatment. Generally, rehabilitation was carried out 3–5 days after the injury, simultaneously with negative pressure surgery or skin grafting, including body positioning and exercises. Our study found that after 4 weeks of standardized rehabilitation, both the TAM and bMHQ scores of the experimental group and the control group improved significantly, which is consistent with our clinical experience.

At the same time, we found no significant difference in TAM and bMHQ scores between the two groups when the negative pressure treatment was finished, and the wound was healed with or without a skin graft. However, after 4 weeks of rehabilitation, the TAM and bMHQ scores in the experimental group were significantly higher than those in the control group, and the excellent and good rates of TAM were also higher. We believe that deep partial-thickness hand burns can be healed within 2–3 weeks due to NPWT, and the difference in hand function between the two groups cannot be seen in this short time. However, the experimental group began to rehabilitate during the early NPWT process and continued it throughout the whole treatment process, which can also be thought of as rehabilitation training extending to the beginning of the treatment. Therefore, their improvement in hand function was superior to that of the control group after adding routine rehabilitation for 4 weeks.

It is not easy to place negative pressure foam to treat deep partial-thickness hand burns. It is necessary to prevent air leakage and to avoid enclosing the whole hand with polyurethane film, which makes it impossible to carry out adequate exercise therapy (27, 28). Therefore, the negative pressure film should be refined as much as possible, and any healthy fingers should be exposed for easy movement when sealed with film. Our experience is that if there are no wounds on the fingers, they should not be covered, and it is essential to keep the thumb in abduction to ensure that the fingers can move freely during the postoperative negative pressure treatment. This allows patients to recover as soon as possible and avoid the function of the fingers being affected by a long immobilization time. Researchers have designed bag-type negative pressure wound therapy, in which the injured hand can be inserted easily to form a sealed bag over just the hand, and the fingers can start exercise training immediately during NPWT (29).

Clinical practice has shown that early application of braces to maintain body position and prevent scar contraction can reduce obvious joint deformities, and the recovery of joint function is obviously better than that of patients who started treatment late, only after joint contraction appeared (30–32). We attempted to assist hand positioning with braces during the NPWT process, even using a thin and low-temperature thermoplastic material (which can be appropriately placed in shape under negative pressure) to maintain thumb abduction and a functional hand position.

Good positioning is one of the best methods to avoid joint contraction and dysfunction. Positioning should start immediately after injury and run throughout the whole treatment process. Positioning should be carried out simultaneously with appropriate joint mobility training to prevent joint contraction and reduced mobility caused by prolonged immobilization. Exercise therapy is the most basic and important treatment method in rehabilitation therapy, including passive exercise and active exercise. Exercise therapy can occur during NPWT until skin grafting. After skin grafting, patients usually gradually transition from passive exercise to active exercise 5–7 days after the operation. Early after injury, patients are encouraged to perform static muscle contraction exercises of immobilized limbs to prevent muscle atrophy and joint rigidity. Early functional training will be accompanied by a certain amount of pain, which requires necessary psychological counseling and spiritual encouragement.

Although the current research data confirm the advantages of the novel therapy combined with early rehabilitation training and NPWT, this study has some limitations. Evaluation of scarring, including scar scores, needs to be added in addition to TAM and bMHQ scores. Furthermore, follow-up time should be further extended to observe the long-term effects of wound repair, including hand function and scarring.

## 5. Conclusion

The application of early rehabilitation training combined with NPWT for the treatment of deep partial-thickness hand burns can effectively improve hand function.

## Data Availability

The raw data supporting the conclusions of this article will be made available by the authors, without undue reservation.
